# Blood glucose and lipids are associated with sarcoidosis: findings from observational and mendelian randomization studies

**DOI:** 10.1186/s12931-023-02663-4

**Published:** 2024-01-22

**Authors:** Yuan Zhan, Jiaheng Zhang, Ruonan Yang, Zhesong Deng, Shanshan Chen, Jie Feng, Jixing Wu, Qian Huang, Yiya Gu, Jungang Xie

**Affiliations:** 1 Department of Respiratory and Critical Care Medicine, National Clinical Research Center of Respiratory Disease, Key Laboratory of Pulmonary Diseases of Health Ministry, Tongji Hospital, Tongji Medical College, Huazhong University of Science and Technology, Wuhan, 430030 Hubei China; 2 Department of Social Medicine and Health Management, School of Public Health, Huazhong University of Science and Technology, Wuhan, Hubei China

**Keywords:** Sarcoidosis, Blood glucose and lipids, Causal association, Propensity score matching, Observational study, Mendelian randomization

## Abstract

**Background:**

Several researches have demonstrated that patients with sarcoidosis accompanied with the abnormality in blood glucose and/or lipids, however, the causal relationship between them remains uncertain. To elucidate the potential association and causality of blood glucose and lipids with sarcoidosis, we conducted a propensity score matching (PSM)-based observational study combined with mendelian randomization (MR) analysis.

**Methods:**

All subjects in this study were retrospectively collected from Tongji Hospital during 2010 and 2023. 1:1 PSM was employed to control the potential confounders as appropriate. Univariable and multivariable logistic regression analyses were performed to estimate the associations of sarcoidosis with fasting glucose, high density lipoprotein cholesterol (HDLC), low density lipoprotein cholesterol (LDLC), total cholesterol (TC), and total triglyceride (TG). The further subtype analysis was also conducted. Afterwards, a bidirectional MR analysis based on public data deeply explored the causality among the 5 candidate traits and sarcoidosis, for which the inverse-variance weighted (IVW) method was utilized as the main inferring approach.

**Results:**

In the observational study, a total number of 756 subjects were enrolled, with 162 sarcoidosis patients and 594 non-sarcoidosis participants, while 160 pairs of subjects were matched after PSM. Multivariable logistic regression analysis indicated that HDLC (OR: 0.151; 95% CI: 0.056–0.408; *P* < 0.001) and TC (OR: 3.942; 95% CI: 2.644–5.877; *P* < 0.001) were strongly associated with sarcoidosis. Subtype analysis showed that low HDLC was independently correlated to risk of lesions in bronchus and lungs, and mediastinal lymph nodes, while high TC was to cervical lymph nodes. In MR analysis, high fasting glucose, low HDLC, and high TC were identified as the causal factors of sarcoidosis.

**Conclusion:**

HDLC and TC had the potential to influence the risk of sarcoidosis, which could be regarded as predictors and may provide new diagnostic and therapeutic targets for sarcoidosis.

**Supplementary Information:**

The online version contains supplementary material available at 10.1186/s12931-023-02663-4.

## Background

Sarcoidosis is a multiple systemic granulomatous inflammation disease with unknown etiology to date, characterized by abnormal immune response to unidentified antigens [1]. Regardless of the variable morbidity in different races (highest in northern Europeans and African Americans), sarcoidosis occurs worldwide and affects all the genders and ages [2, 3], while the global age-standardized rates of incidence and prevalence are increasing [4]. The tissues and organs affected by sarcoidosis can be alone or multiple and include lung, lymph node, skin, eye, liver, kidney, nervous system, heart, and/or muscle [5]. The clinical manifestation and course of sarcoidosis also vary considerably, ranging from asymptomatic stage, to acute onset or exacerbation, to chronic progressive illness with many unspecific symptoms, resulting in difficult diagnosis [5]. Systemic treatment with corticosteroids is still the mainstay for sarcoidosis, while the second-line therapy is immunosuppressants such as methotrexate and azathioprine [3]. Suffering the side effects of corticosteroids and/or immunosuppressants, some patients still gradually develop irreversible complications such as pulmonary fibrosis and heart failure, which seriously affect the quality and length of life [6]. Therefore, new diagnostic and therapeutic targets are expected to improve the situations of patients.

Blood contains numerous kinds of metabolites [7], among which blood glucose and lipids are the most common clinical traits. Blood glucose and lipids have already been found to be associated with kinds of idiopathic inflammatory diseases such as rheumatoid arthritis [8] and inflammatory bowel disease [9, 10], but their roles in the onset of sarcoidosis are not yet clear. The metabolic disorders related to sarcoidosis found so far include obesity [2, 11], diabetes [12] and dyslipidemia [13], which conforms to our clinical experience that sarcoidosis patients tend to have more problems with blood glucose and lipids. The changes of glucose [14, 15] and lipid [16] metabolites can also be observed in sarcoidosis patients’ blood. However, the reliability of these conclusions is limited by conventional observational research design, and the causality between metabolic disorder and sarcoidosis is still confusing. Further studies are necessary to clarify the relationships between blood metabolites and sarcoidosis, which might have an important enlightenment to basic research and clinical practice to help in the diagnosis and treatment of sarcoidosis.

Mendelian randomization (MR) is a well-established tool utilizing selective genetic instrumental variables (IVs) to evaluate the causality without many of the common biases affecting the validity of conclusions [17]. In this study, the retrospective observational study about Asian sarcoidosis cases from our clinical data was combined with MR analysis based on European large-scale genome-wide association study (GWAS) data to deeply explore the association of sarcoidosis with blood glucose and lipids.

## Methods

### Observational study design

A propensity score matching (PSM)-based retrospective case-control study was performed using the clinical data from the Tongji Hospital, Tongji Medical College, Huazhong University of Science and Technology. The inclusion criteria for sarcoidosis cases were as below: (1) “sarcoidosis” was included in the discharge diagnosis; (2) the diagnosis was confirmed by pathological examination; (3) the possibility of other granulomatous diseases such as tuberculosis was excluded; (4) there was no malignant or severe underlying illness; and (5) there was no history of corticosteroid treatment 6 months before admission. And the non-sarcoidosis subjects were from the Health Examination Center of Tongji Hospital.

Five traits including fasting glucose, high density lipoprotein cholesterol (HDLC), low density lipoprotein cholesterol (LDLC), total cholesterol (TC), and total triglyceride (TG) were selected as “blood glucose and lipid traits” in this study due to their clinical accessibility, while the other 16 traits were used to comprehensively represent the pathophysiological conditions of participants.

### Study participants

**Fig. 1:**
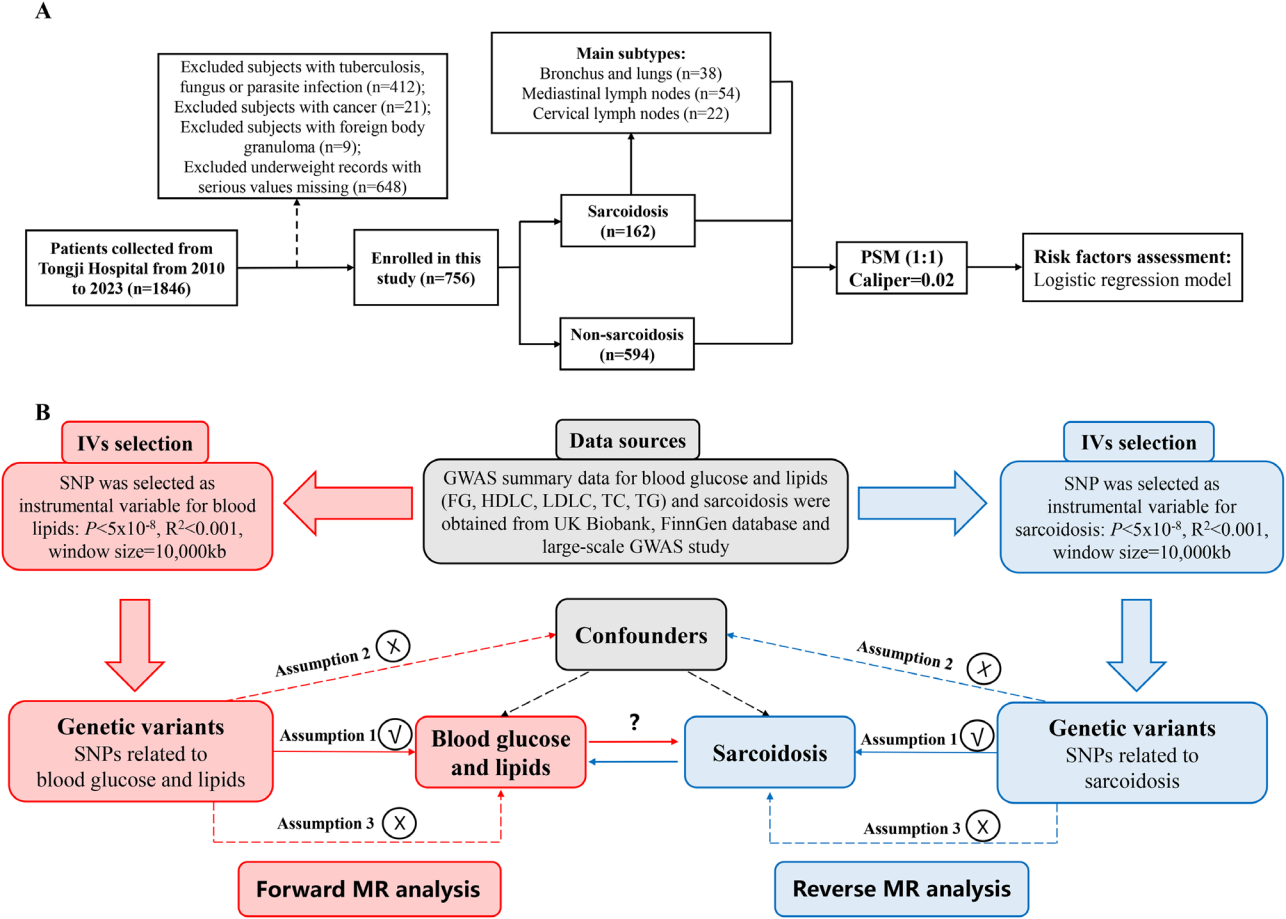
Design flowchart of observational and mendelian randomization study. **(A)** Flowchart of patient determination in the observational study. **(B)** Flowchart of bidirectional MR study. PSM, propensity score matching. FG, fasting glucose. HDLC, high density lipoprotein cholesterol. LDLC, low density lipoprotein cholesterol. TC, total cholesterol. TG, total triglyceride. MR, mendelian randomization. GWAS, genome-wide summary association study. IVs, instrumental variables. SNP, single nucleotide polymorphism

As shown in Fig. 1A, 1,846 adult participants (> 18 years old) ranging from January 2010 to August 2023 were preliminarily collected from Tongji Hospital, including 846 subjects with suspected sarcoidosis and 1,000 non-sarcoidosis controls from the Health Examination Center based on simple random sampling. After criteria screening and quality control, a total of 756 cases were enrolled in this study, consisting of 162 cases with sarcoidosis and 594 cases without sarcoidosis. The retrospective review of the medical records was approved by the Ethics Committee of Tongji Hospital, Tongji Medical College, Huazhong University of Science and Technology and was carried out according to the principles expressed in the Declaration of Helsinki. Due to the observational character of this study, the patient’s consent to participate was exempted and registration at a clinical trial registry was not required.

### Propensity score matching

PSM is a statistical method commonly used to reduce the confounding bias from observational study, which matches control group with case group to establish a “new” control group after discarding outlier subjects [18]. By matching the demographic characteristics and comorbidities, 1:1 PSM was performed based on the estimated propensity score for all the sarcoidosis cases. Furthermore, in order to inspect blood glucose and lipid traits in the main subtypes of sarcoidosis, 1:1 PSM including all the factors except the one trait of interest was performed respectively. The caliper distance for all the PSM was 0.02.

### Statistical analysis

Continuous variables with normal distribution, continuous variables with skewed distribution, and enumerable variables were presented as the average value with standard deviation, the median with quartile, and the number with percentage, respectively. Differences between groups were tested by t test, Wilcoxon sign rank sum test, or chi-square test. Univariable and stepwise multivariable logistic regression analysis including all the factors with significant differences was applied before and after PSM, for which collinearity diagnosis had been carried out.

All the analysis mentioned above was performed with IBM SPSS Statistics, Version 26.0 (Armonk, NY: IBM Corp). The two-sided *P*-value < 0.05 was considered significant.

### Bi-directional MR design

Two-sample MR analysis was bilaterally performed to infer causal associations of sarcoidosis with blood glucose and lipid traits. MR is a widely-accepted statistical method to evaluate the causality of exposure/intermediate factors with outcomes, characterized by relatively high evidence strength without much influence of confounders [17, 19]. As depicted in Fig. 1B, a valid MR need to satisfy three fundamental assumptions as follow: (1) as IVs, instrumental single-nucleotide polymorphisms (SNPs) should be strongly associated with the exposure; (2) the IVs can not be associated with confounders; (3) the IVs must be associated with the outcome only via the exposure [17]. Ethical approval was exempted due to the public availability of data for this MR study.

### SNPs sources and IV selection

Summary-level data for sarcoidosis were derived from the FinnGen with 2,046 cases and 215,712 controls (GWAS ID: finn-b-D3_SARCOIDOSIS), which can be publicly accessed via the IEU OpenGWAS project (https://gwas.mrcieu.ac.uk/). SNPs closely associated with the 5 kinds of candidate traits were also obtained from the IEU OpenGWAS project. All participants in MR analysis were of European ancestry, minimized the potential bias owing to the population heterogeneity. The summary of studies included is listed in Table S1.

SNPs with genome-wide significance were selected as IVs with *P* < 5 × 10^− 8^. The threshold of 5 × 10^− 8^ is widely recognized as the criterion for declaring genome-wide association significance for common variants with a minor allele frequency no less than 5% in European ancestry populations [20]. To avoid the linkage disequilibrium (LD) bias, the LD threshold was set as R^2^ < 0.001, with the window size of 10,000 kb. The F statistic was used to evaluate the strength of each SNP. Generally, F > 10 was regarded as strong association. The specific information for every IV was summarized in Supplementary material 1 and 2. All the SNPs were no less than 3, and the minimum value of F statistic was 24.5, providing guarantees for the reliability of this MR analysis.

### MR estimates and sensitivity analysis

For MR analysis, the random-effect inverse variance weighted (IVW) method was employed as the primary estimate approach, while MR-Egger, Weighted median, Simple mode and Weighted mode were utilized as complementary methods. The results were shown as odds ratios (OR) and 95% confidence intervals (CI).

The heterogeneity between individual genetic variations was estimated by the Cochran’s Q test. Then, the intercept obtained from the MR-Egger regression model was calculated to test the horizontal pleiotropy introduced by unknown confounding factors. Finally, the leave-one-out analysis was conducted to determine the stability of MR results via successively excluding each SNP. All the analysis in MR was performed in R (version 4.0.3) with R package “TwoSampleMR” [21]. All the results were considered statistically significant at *P* < 0.05.

## Results

### Characteristics of patients in observational study

The baseline information of all the 756 patients were reported at the left half of Table 1. The demographic characteristics between case group and control group were significantly different while case group had more uveitis and control group had more hypertension, which might cause confounding bias. In order to control the confounding factors from demographic characteristics and comorbidities, 1:1 PSM was preformed and 160 pairs of subjects were obtained as shown at the right half of Table 1. All the 5 kinds of blood glucose and lipid traits kept significant differences after PSM, which preliminarily confirmed our hypothesis. Meanwhile, the differences were also significant in some other traits such as blood potassium and globulin.

**Table 1:** Baseline information of enrolled patients before or after propensity score matching

	Before matching			After matching		
	Sarcoidosis (*n* = 162)	Non-sarcoidosis (*n* = 594)	*P* value	Sarcoidosis (*n* = 160)	Non-sarcoidosis (*n* = 160)	*P* value
**Demographic characteristics**						
Sex			< 0.001			> 0.999
Male	53 (32.7%)	311 (52.4%)		53 (33.1%)	53 (33.1%)	
Female	109 (67.3%)	283 (47.6%)		107 (66.9%)	107 (66.9%)	
Age (years)	48.79 (11.79)	46.05 (13.03)	0.016	48.54 (11.65)	49.08 (11.73)	0.681
Weight (kg)	61.54 (11.00)	64.18 (11.32)	0.008	61.57 (11.06)	61.95 (10.26)	0.752
Smoking history	19 (11.7%)	133 (22.4%)	0.003	19 (11.9%)	22 (13.8%)	0.738
**Comorbidities**						
Hypertension	13 (8.0%)	97 (16.3%)	0.008	13 (8.1%)	17 (10.6%)	0.566
Coronary disease	6 (3.7%)	31 (5.2%)	0.540	6 (3.8%)	6 (3.8%)	> 0.999
Fatty liver	14 (8.6%)	42 (7.1%)	0.500	12 (7.5%)	13 (8.1%)	> 0.999
Diabetes	18 (11.1%)	66 (11.1%)	> 0.999	18 (11.3%)	22 (13.8%)	0.613
Uveitis	4 (2.5%)	1 (0.2%)	0.009	4 (2.5%)	0 (0.0%)	0.123
**Blood glucose and lipid traits**						
Fasting glucose (mmol/L)	5.87 (5.02–6.65)	5.40 (4.90-6.00)	< 0.001	5.87 (5.03–6.66)	5.40 (4.85-6.00)	< 0.001
HDLC (mmol/L)	1.09 (0.34)	1.21 (0.34)	< 0.001	1.07 (0.89–1.21)	1.24 (1.03–1.50)	< 0.001
LDLC (mmol/L)	2.82 (2.33–3.14)	2.41 (1.90–2.88)	< 0.001	2.82 (2.32–3.15)	2.46 (1.99–2.83)	< 0.001
TC (mmol/L)	4.65 (4.10–5.43)	3.92 (3.36–4.46)	< 0.001	4.65 (4.09–5.42)	4.02 (3.49–4.56)	< 0.001
TG (mmol/L)	1.65 (1.16–2.39)	1.16 (0.77–1.80)	< 0.001	1.65 (1.16–2.40)	1.11 (0.80–1.86)	< 0.001
**Other blood traits**						
Blood potassium (mmol/L)	3.99 (0.29)	4.14 (0.28)	< 0.001	3.99 (0.29)	4.14 (0.27)	< 0.001
Blood calcium (mmol/L)	2.34 (0.13)	2.30 (0.10)	0.001	2.33 (2.26–2.40)	2.30 (2.25–2.37)	0.058
ALT (U/L)	19.00 (14.00-24.20)	17.07 (12.00-24.76)	0.075	19.21 (14.00-24.52)	16.00 (12.10-24.76)	0.067
AST (U/L)	20.91 (18.00-23.50)	19.00 (16.00–24.00)	0.004	20.91 (18.00-23.75)	19.00 (16.25-24.00)	0.027
GGT (U/L)	28.33 (19.00–49.00)	21.35 (14.83–32.50)	< 0.001	28.33 (19.00-50.50)	20.73 (14.31–28.78)	< 0.001
ALP (U/L)	73.00 (61.00-92.67)	69.00 (58.25-82.00)	0.010	73.14 (61.00-92.67)	69.63 (58.00-83.10)	0.083
ALB (g/L)	42.71 (3.64)	43.37 (3.73)	0.045	42.67 (3.65)	43.60 (3.24)	0.016
Globulin (g/L)	30.58 (5.10)	27.64 (3.83)	< 0.001	29.95 (27.35–33.27)	27.62 (25.33–30.16)	< 0.001
TBil (umol/L)	9.18 (7.10–12.40)	8.69 (6.40-11.73)	0.046	9.23 (7.10–12.40)	8.28 (6.60–11.00)	0.034
UA (umol/L)	310.00 (257.00-396.10)	306.40 (258.73-371.86)	0.254	310.00 (256.60-396.35)	287.00 (236.41-341.88)	0.001
Urea (mmol/L)	5.39 (4.30–6.65)	5.10 (4.33–6.05)	0.095	5.34 (4.30–6.68)	5.13 (4.39–6.07)	0.283
Creatinine (umol/L)	68.00 (57.00–79.00)	71.00 (58.00-83.29)	0.221	68.00 (57.00-79.50)	64.00 (55.13–76.32)	0.085
RBC (10^12/L)	4.52 (0.52)	4.47 (0.58)	0.305	4.53 (0.52)	4.43 (0.58)	0.127
Hb (g/L)	132.64 (15.12)	134.12 (19.20)	0.300	132.75 (15.17)	130.68 (18.54)	0.274
PLT (10^9/L)	223.38 (190.00-272.86)	214.80 (182.00-252.75)	0.032	223.38 (190.25-273.43)	223.75 (181.08-250.25)	0.149
WBC (10^9/L)	5.70 (4.47–6.62)	6.19 (5.12–7.31)	< 0.001	5.68 (4.44–6.61)	5.89 (5.01–7.06)	0.052

N (%), mean (SD), median (25-75%). HDLC, high density lipoprotein cholesterol. LDLC, low density lipoprotein cholesterol. TC, total cholesterol. TG, total triglyceride. ALT, alanine amino transferase. AST, aspartate amino transferase. GGT, gamma glutamyl transferase. ALP, alkaline phosphatase. ALB, albumin. TBil, total bilirubin. UA, uric acid. RBC, red blood cell. Hb, hemoglobin. PLT, platelet. WBC, white blood cell

The distribution of sarcoidosis lesions was counted in Table S2. According to the results of pathological biopsy, most of the lesions were found in bronchus and lungs (*n* = 38, 23.5%), mediastinal lymph nodes (*n* = 54, 33.3%), and cervical lymph nodes (*n* = 22, 13.6%), while 20.4% of the cases had more than one positive lesion.

### Association of sarcoidosis with blood glucose and lipids

To explore the associations between sarcoidosis and blood glucose or lipids, univariable and multivariable logistic regression analysis was performed respectively. In the univariable logistic regression analysis, all the 5 kinds of blood glucose and lipid traits showed significant associations with sarcoidosis before and after PSM (Table 2). In the multivariable logistic regression analysis, collinearity diagnosis showed no significances among all covariables included (Table S3 and S4) and results demonstrated significant association for fasting glucose (OR: 1.449; 95%CI: 1.196–1.756; *P* < 0.001), HDLC (OR: 0.076; 95%CI: 0.033–0.176; *P* < 0.001), and TC (OR: 4.606; 95%CI: 3.332–6.366; *P* < 0.001) with sarcoidosis, but only HDLC and TC kept significance after PSM (Table 2). Compared to the matched control group, sarcoidosis was correlated with lower HDLC (OR: 0.151; 95%CI: 0.056–0.408; *P* < 0.001) and higher TC (OR: 3.942; 95%CI: 2.644–5.877; *P* < 0.001). The correlations of sarcoidosis with LDLC and TG were rejected in the part of observational study.

**Table 2:** Association analysis of blood glucose and lipid traits with sarcoidosis

	Before matching				After matching			
	Univariable OR (95% CI)	*P* value	Multivariable OR (95% CI)	*P* value	Univariable OR (95% CI)	*P* value	Multivariable OR (95% CI)	*P* value
Fasting glucose	1.501 (1.307–1.725)	< 0.001	1.449 (1.196–1.756)	< 0.001	1.546 (1.265–1.889)	< 0.001	-	-
HDLC	0.312 (0.175–0.559)	< 0.001	0.076 (0.033–0.176)	< 0.001	0.181 (0.085–0.386)	< 0.001	0.151 (0.056–0.408)	< 0.001
LDLC	2.863 (2.135–3.839)	< 0.001	-	-	2.529 (1.761–3.631)	< 0.001	-	-
TC	3.463 (2.690–4.460)	< 0.001	4.606 (3.332–6.366)	< 0.001	2.661 (1.980–3.578)	< 0.001	3.942 (2.644–5.877)	< 0.001
TG	1.525 (1.310–1.776)	< 0.001	-	-	1.601 (1.269–2.019)	< 0.001	-	-

Multivariable OR was calculated by Binary logistic regression with a forward stepwise selection approach (level for selection: *P* = 0.05, and level for elimination: *P* = 0.10). The multivariable logistic regression model encompassed all the traits with significant difference before or after matching in baseline information. HDLC, high density lipoprotein cholesterol. LDLC, low density lipoprotein cholesterol. TC, total cholesterol. TG, total triglyceride. OR, odds ratio. CI, confidence interval

**Fig. 2:**
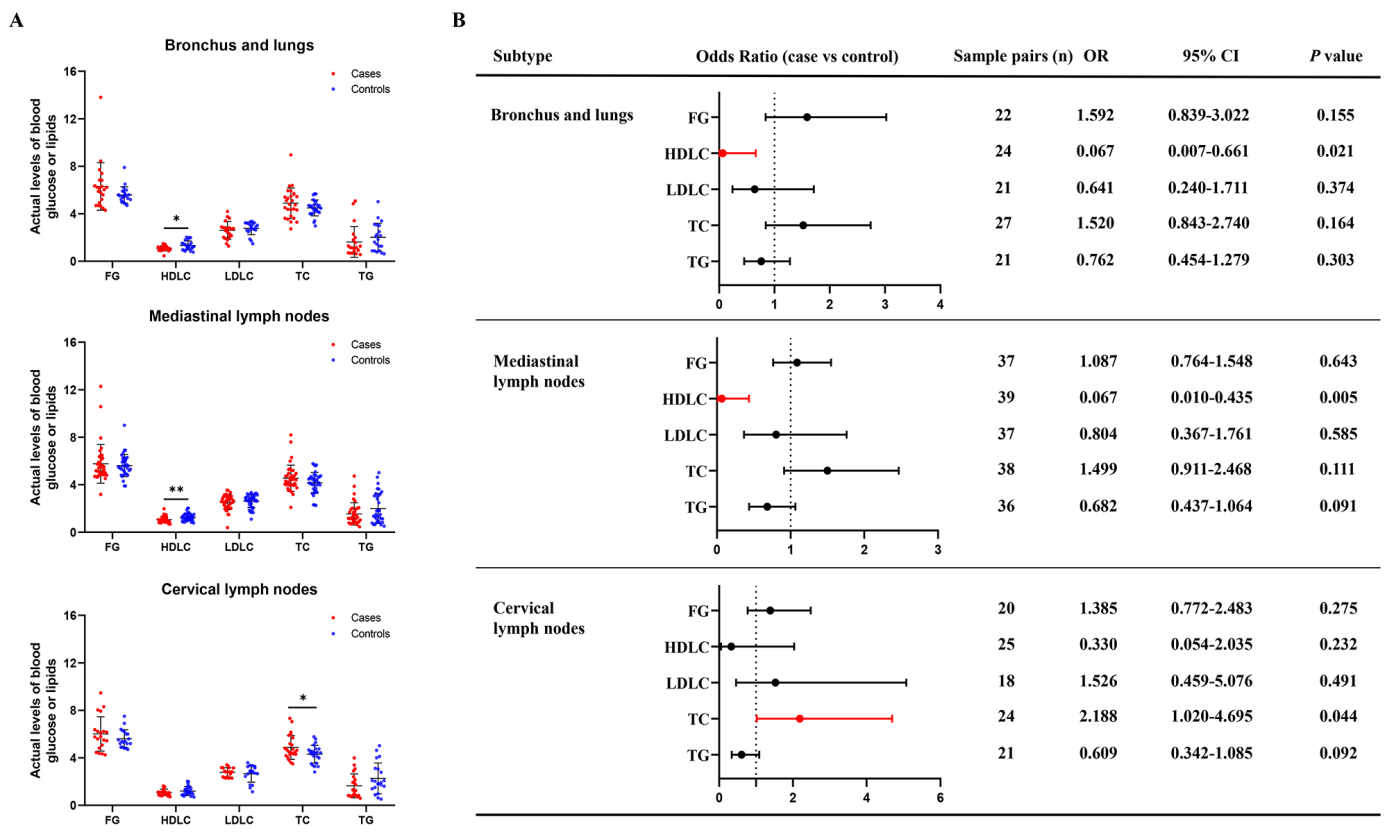
Association analysis of blood glucose and lipids with main subtypes of sarcoidosis. Risks of blood glucose and lipids on three subtypes of sarcoidosis were estimated, including bronchus and lungs, mediastinal lymph nodes, and cervical lymph nodes. PSM was respectively conducted among various subgroups, with all covariates balanced excluding detected indicator. **(A)** The actual levels of blood glucose or lipids in three subtypes after PSM. **(B)** Risk assessment between various blood glucose or lipids and different subtypes using univariable logistic regression model after PSM. PSM, propensity score matching. FG, fasting glucose. HDLC, high density lipoprotein cholesterol. LDLC, low density lipoprotein cholesterol. TC, total cholesterol. TG, total triglyceride. OR, odds ratio. CI, confidence interval

To further examine the association between these blood metabolites and subtypes of sarcoidosis, 1:1 PSM including all the other factors was performed respectively for the 5 candidate traits in 3 main lesions. Figure 2A depicted the distribution of data after PSM, and statistical test confirmed the significant differences of HDLC in cases of mediastinal lymph nodes, bronchus and lungs, and TC in cases of cervical lymph nodes. Figure 2B showed the results of logistic regression analysis, in which HDLC in cases of bronchus and lungs (OR: 0.067; 95%CI: 0.007–0.661; *P* = 0.021) and mediastinal lymph nodes (OR: 0.067; 95%CI: 0.010–0.435; *P* = 0.005), and TC in cases of cervical lymph nodes (OR: 2.188; 95%CI: 1.020–4.695; *P* = 0.044) showed significant associations.

### Main analysis for MR

**Fig. 3:**
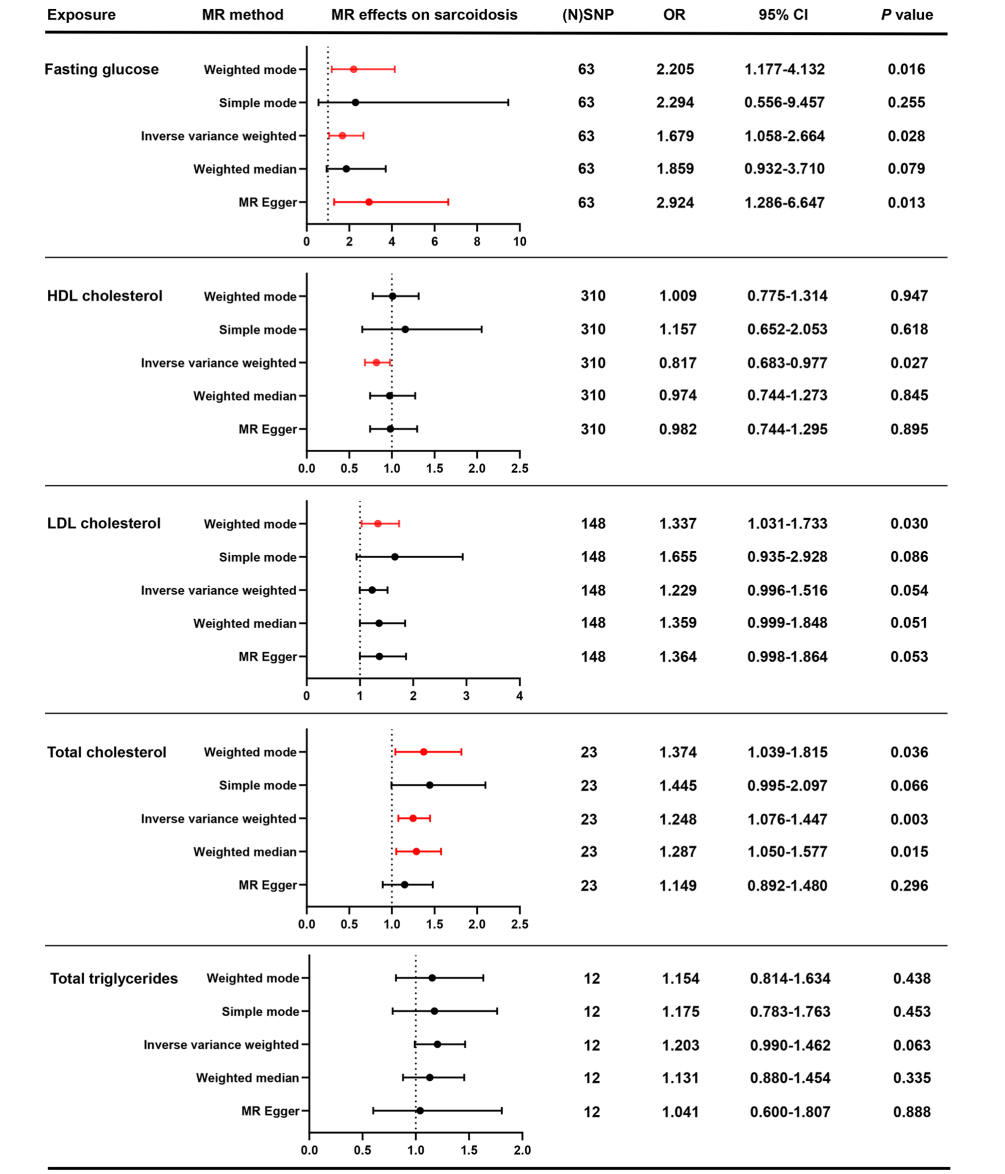
Forest plots for the causal associations of blood glucose and lipids with sarcoidosis. The forest plots showed the genetic associations of blood glucose and lipids with sarcoidosis. MR, mendelian randomization. SNP, single nucleotide polymorphism. OR, odds ratio. CI, confidence interval. HDLC, high density lipoprotein cholesterol. LDLC, low density lipoprotein cholesterol

As indicated by the results of the main method Random-effect IVW, fasting glucose, HDLC, and TC showed significant causality on sarcoidosis (Fig. 3). Specifically, higher fasting glucose (OR: 1.679; 95%CI: 1.058–2.664; *P* = 0.028) and TC (OR: 1.248; 95%CI: 1.076–1.447; *P* = 0.003) are accompanied with higher risk of sarcoidosis, while higher HDLC (OR: 0.817; 95%CI: 0.683–0.977; *P* = 0.027) predicts less occurrence of sarcoidosis. The weighted mode and MR Egger supported the result of fasting glucose, while the weighted mode and weighted median supported the result of TC. Additionally, the scatter plots and forest plots also demonstrated the consistent results (Fig. S2 and S3). In this study, the causal relationships of LDLC and TG on sarcoidosis were not approved. Reversely, sarcoidosis could not be considered as the upstream factors for the 5 traits with the negative results in Fig. S1.

### Sensitivity analysis for MR

Cochran’s Q test revealed the heterogeneity between individual genetic variations for all the 10 groups of SNPs, of which some traits showed significant heterogeneity (Table 3 and S5). The large sizes of these IVs explained part of the heterogeneity, and it should be noted that the random-effect IVW approach utilized in this research accommodated for the existence of heterogeneity across the IVs.

**Table 3:** Heterogeneity and pleiotropy tests of the forward mendelian randomization analysis

Test	Exposure	Method	Effect size	*P* value
Heterogeneity	Fasting glucose	Cochran’s Q test	78.8 (Q_MR Egger_)	0.063
			82.0 (Q_IVW_)	0.045
	HDLC	Cochran’s Q test	399.7 (Q_MR Egger_)	0.000
			403.4 (Q_IVW_)	0.000
	LDLC	Cochran’s Q test	187.3 (Q_MR Egger_)	0.012
			188.3 (Q_IVW_)	0.012
	TC	Cochran’s Q test	10.9 (Q_MR Egger_)	0.964
			11.5 (Q_IVW_)	0.966
	TG	Cochran’s Q test	7.5 (Q_MR Egger_)	0.679
			7.8 (Q_IVW_)	0.733
Pleiotropy	Fasting glucose	MR-Egger regression	-0.016 (Egger regression intercept)	0.116
	HDLC	MR-Egger regression	-0.007 (Egger regression intercept)	0.091
	LDLC	MR-Egger regression	-0.005 (Egger regression intercept)	0.377
	TC	MR-Egger regression	0.013(Egger regression intercept)	0.439
	TG	MR-Egger regression	0.018 (Egger regression intercept)	0.595

MR, Mendelian randomization. IVW, inverse variance weighted. HDLC, high density lipoprotein cholesterol. LDLC, low density lipoprotein cholesterol. TC, total cholesterol. TG, total triglyceride. HDLC, high density lipoprotein cholesterol. LDLC, low density lipoprotein cholesterol. TC, total cholesterol. TG, total triglyceride

No evidence of horizontal pleiotropy was found according to the MR-Egger regression intercept, which fulfilled the last 2 assumptions of MR analysis (Table 3 and S5). The leave-one-out analysis denied that the positive results were largely decided by some extreme values (Fig. S4).

## Discussion

Similar to many other idiopathic inflammation diseases, environmental exposures on genetically susceptible individuals are believed to cause sarcoidosis [3, 22]. Cued by racial differences and family aggregation, genetical susceptibility might be essential for sarcoidosis, most of which is related to human leukocyte antigen (HLA) [22, 23]. In terms of environmental exposures, insecticides, agricultural employment, microbial bioaerosols, metal contact, and high humidity have been pointed as potential trigger factors [24, 25], although some of the conclusions are still debatable and for some patients none of the known exposures can be found. Besides, the patients’ common features have been summarized as the risk factors of sarcoidosis, which includes obesity and specific infection such as tuberculosis [2, 26], while female hormones might be a protective factor and the role of tobacco smoking remains controversial [3].

To broaden our understanding of sarcoidosis, we retrospectively analyzed the clinical data about 5 kinds of blood glucose and lipid traits (fasting glucose, HDLC, LDLC, TC, and TG) in patients with sarcoidosis. Through statistical analysis for PSM-based case-control study, we found that HDLC and TC were strongly associated with sarcoidosis, which was basically verified in the main subtypes. And then, MR analysis was performed to further clarify the causality of blood glucose and lipids with sarcoidosis, by which fasting glucose, HDLC, and TC were recognized as the upstream factors of sarcoidosis, but not vice versa. In all, HDLC and TC can be regarded as important risk factors of sarcoidosis, which indicates potential functions of blood metabolites and deeper understanding of sarcoidosis.

The association between sarcoidosis and diabetes mellitus has already been pointed out by a meta-analysis of 19 studies [12], which suggested that the level of blood glucose might be related to risk of sarcoidosis. Correspondingly, MR analysis in our study concluded that it was blood glucose to be the causal role in this relationship. Many studies are prone to take chronic inflammation as the contributor to type 2 diabetes [27, 28], but hyperglycemia can also worsen systematic inflammation with the formation of advanced glycation end products (AGEs) [29] and the immunity training in macrophages [30]. While impairing the anti-infection function of immune system [31], hyperglycemia is believed to exacerbate autoimmunity through Th17 cells [32]. In fact, not only glucose itself, but many of its blood metabolites such as pyruvate, succinate, and lactate also undergo changes in sarcoidosis [14, 15], which highlighted the importance of glucose metabolism disorder in sarcoidosis. However, in our case-control study, blood glucose lost its significance for sarcoidosis in multivariable logistic regression analysis after PSM. One of the possible reasons was that the observational study combined with PSM controlled the confounding bias more effectively [18] and might tend to underestimate the association between blood traits and sarcoidosis. And our negative results further emphasized the racial heterogeneity in the pathogenesis of sarcoidosis [3], which might be part of the contributors to the variable prevalence of DM in sarcoidosis of different regions [12] and needs to be taken into account in future researches.

Although the related studies are limited, a Serbian case-control study has found more dyslipidemia in sarcoidosis, which concluded that TG was significantly higher whereas HDLC was significantly lower in sarcoidosis patients [13]. Besides, levels of n-3 poly-unsaturated fatty acids (PUFAs) and n-6 PUFAs could predict sarcoidosis and its organ involvement [16]. In mechanism, dyslipidemia can accelerate inflammatory and autoimmune responses through cytokines [33] and oxidative stress [34]. Specifically, HDLC reduces inflammation in multiple cell types including endothelial cells and macrophages by several mechanisms involving apolipoprotein A-I and cholesterol efflux [35], while hypercholesterolaemia leads to cholesterol accumulation and metabolism reprogramming in immune cells and promotes inflammatory responses [36, 37], which may account for the upstream roles of HDLC and TC in sarcoidosis found in this study. The interaction between blood lipids and immune cells found so far can be introduced into the basic research of sarcoidosis, which in turn can promote deeper understanding of dyslipidemia in inflammatory diseases.

Due to the multiple organ involvement, unspecific symptoms, variable clinical courses, and the lack of sensitive biomarkers, the diagnosis of sarcoidosis is difficult and depends on adequate clinical experience, typical imaging presentations, and pathological biopsy if necessary [5]. Based on this study, blood glucose and lipid traits can be developed as part of the diagnostic evidence for sarcoidosis. The current treatment for sarcoidosis, such as corticosteroids and immunosuppressants, is not individualized and their efficacy is unsatisfied for some patients [3, 6]. Considering that patients with sarcoidosis were more likely to be prescribed with corticosteroid, what is even worse, one of the major side effects is just the metabolic disorder of glucose and lipids [38, 39]. Therefore, the therapy targeting blood glucose and lipids might be necessary and effective for sarcoidosis patients.

As one of the major health issues in modern world, metabolic syndrome, characterized by abdominal obesity, insulin resistance, hypertension, and dyslipidemia, is increasingly prevalent worldwide [40]. This might contribute to the increasing global age-standardized rates of incidence and prevalence of sarcoidosis [4] to some extent since obesity and abnormal blood glucose and lipids are the risk factors of sarcoidosis. Epidemiologically, it is necessary to strengthen the screening work of high-risk groups and help them maintain healthy blood glucose and lipids, which can be beneficial for early prevention and treatment of sarcoidosis.

The powerful results of this study were based on the combination of PSM-based retrospective case-control study and MR analysis [17, 18]. Data from two racial groups also ensured the consistency of conclusions. However, it should be noted that this study was limited to the risk of sarcoidosis and further researches are needed for the relationship of clinical presentations and prognosis with blood glucose and lipids in sarcoidosis, which is necessary for the therapeutic value of controlling blood glucose and lipids on sarcoidosis. In addition, the association and causality of blood glucose and lipids with sarcoidosis needs more basic researches, which can help us better understand the role of blood metabolites in immune diseases and systemic inflammation to find out new diagnostic and therapeutic targets and improve the curative effect and life quality of patients.

## Conclusions

In this study, the association and causality of 5 kinds of blood glucose and lipid traits (fasting glucose, HDLC, LDLC, TC, and TG) with sarcoidosis was evaluated by retrospective observational study and MR analysis, respectively. Fasting glucose, HDLC, and TC were identified as the upstream factors of sarcoidosis, among which HDLC and TC showed strong association with sarcoidosis in PSM-based case-control study. This finding preliminarily indicated the importance of blood glucose and lipids in the etiopathogenesis of sarcoidosis and can direct further researches about the basic mechanism and therapeutic value of blood glucose and lipids on sarcoidosis.

### Electronic supplementary material

Below is the link to the electronic supplementary material.

Supplementary Material 1

Supplementary Material 2

Supplementary Material 3

## Data Availability

No datasets were generated or analysed during the current study.

## Abbreviations

CI: confidence intervals
DM: diabetes mellitus
GWAS: genome-wide association study
HDLC: high density lipoprotein cholesterol
IV: instrumental variable
IVW: inverse-variance weighted
LD: linkage disequilibrium
LDLC: low density lipoprotein cholesterol
MR: mendelian randomization
OR: odds ratios
PSM: propensity score matching
PUFAs: poly-unsaturated fatty acids
SNP: single-nucleotide polymorphism
TC: total cholesterol
TG: total triglyceride
