# Supplementary material for: Blood glucose and lipids are associated with sarcoidosis: findings from observational and mendelian randomization studies
Source: Respir Res. 2024 Jan 22;25:50. doi: 10.1186/s12931-023-02663-4 (PMC10804582; doi:10.1186/s12931-023-02663-4)
Supplement: Supplementary file 3 — Supplementary Material 3 [file 12931_2023_2663_MOESM3_ESM.docx]

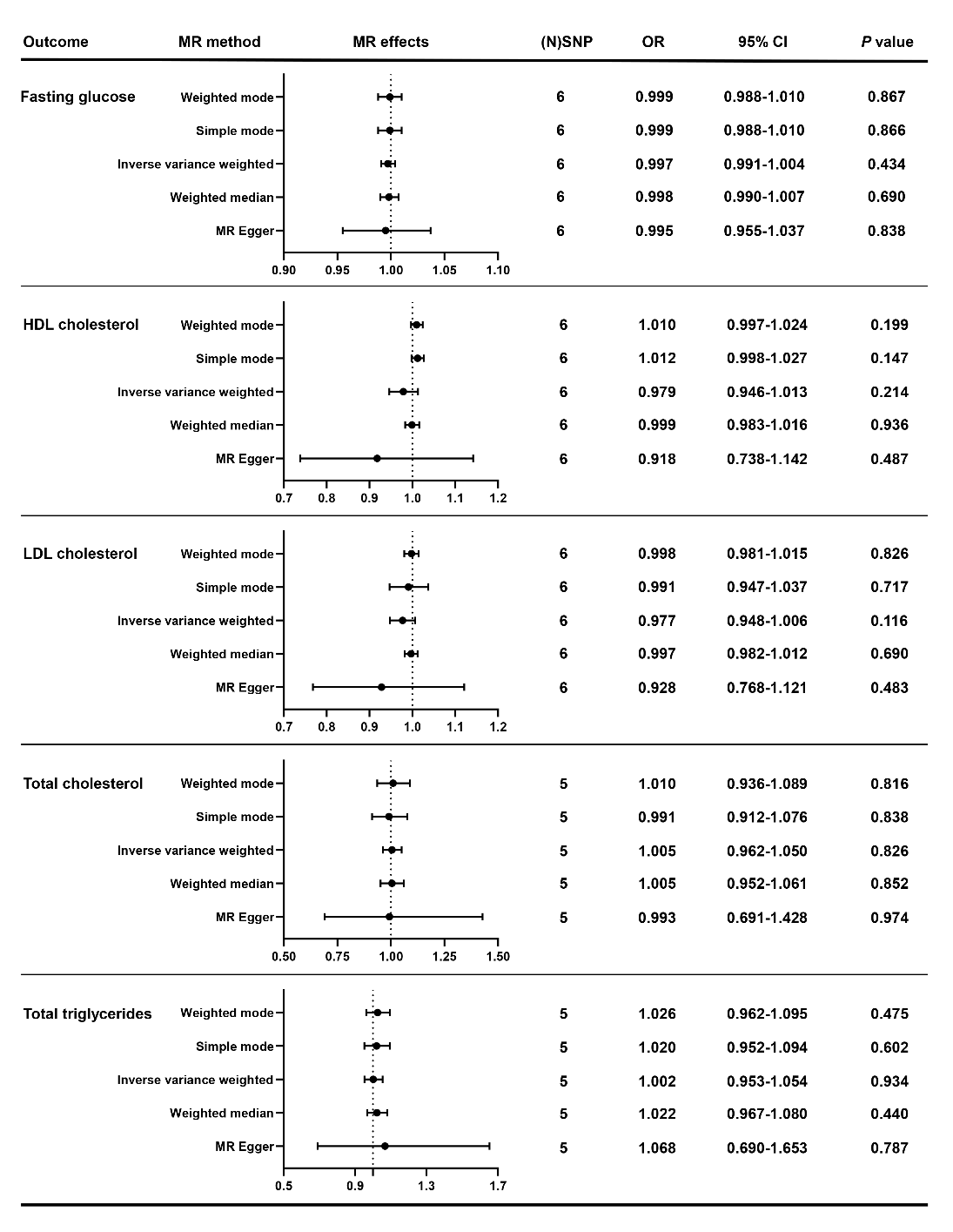


**Fig. S1.** Forest plots for the reverse MR results.

The forest plots showed the causal effects of sarcoidosis on blood glucose and lipids. MR, mendelian randomization. SNP, single nucleotide polymorphism. OR, odds ratio. CI, confidence interval. HDLC, high density lipoprotein cholesterol. LDLC, low density lipoprotein cholesterol.


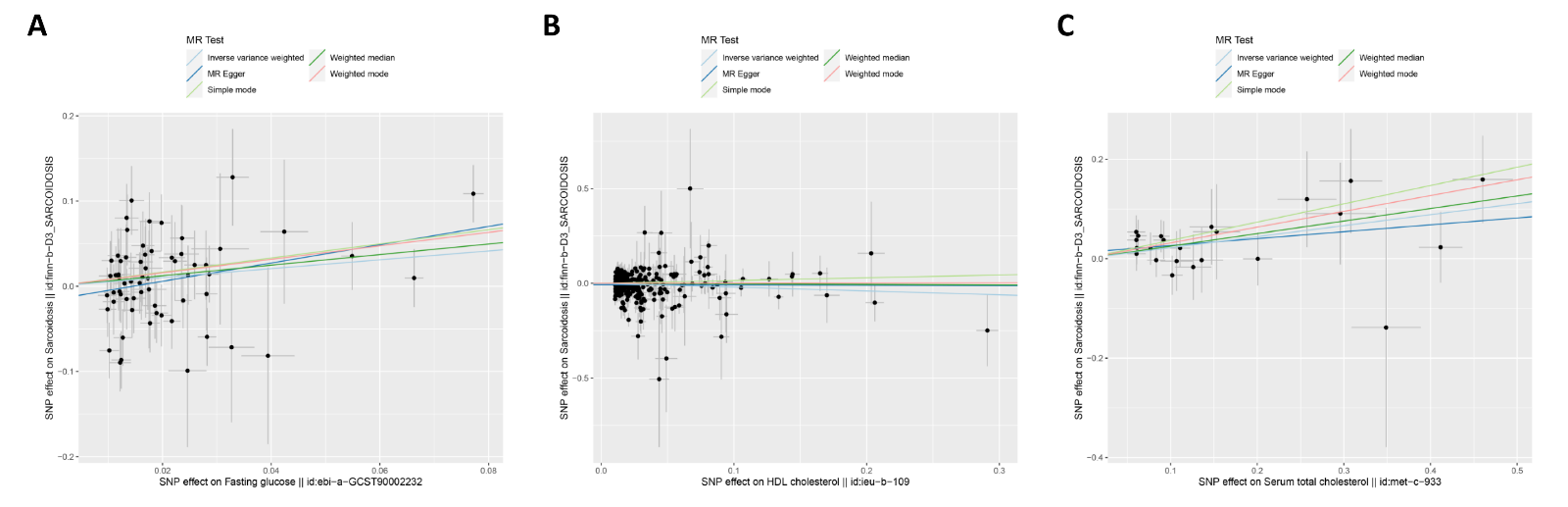


**Fig. S2.** Scatter plots for the causality of fasting glucose, HDLC and TC with sarcoidosis.

The scatter plots showed the genetic associations of fasting glucose **(A)**, HDLC **(B)**, and TC **(C)**. MR, mendelian randomization. SNP, single nucleotide polymorphism. HDLC, high density lipoprotein cholesterol. TC, total cholesterol.


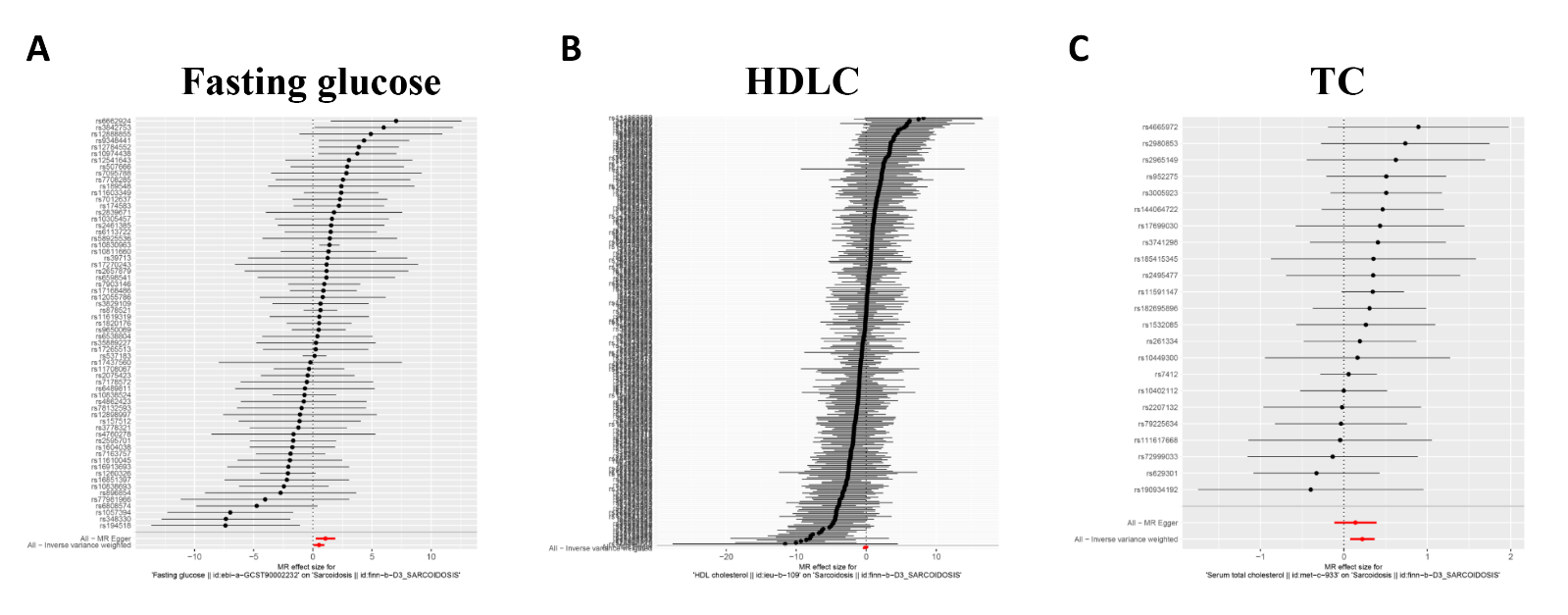


**Fig. S3.** The causal effect of individual SNP in MR analyses.

The forest plots showed the MR effect size of each SNP on sarcoidosis respectively via fasting glucose (A), HDLC (B), and TC (C). MR, mendelian randomization. SNP, single nucleotide polymorphism. HDLC, high density lipoprotein cholesterol. TC, total cholesterol.


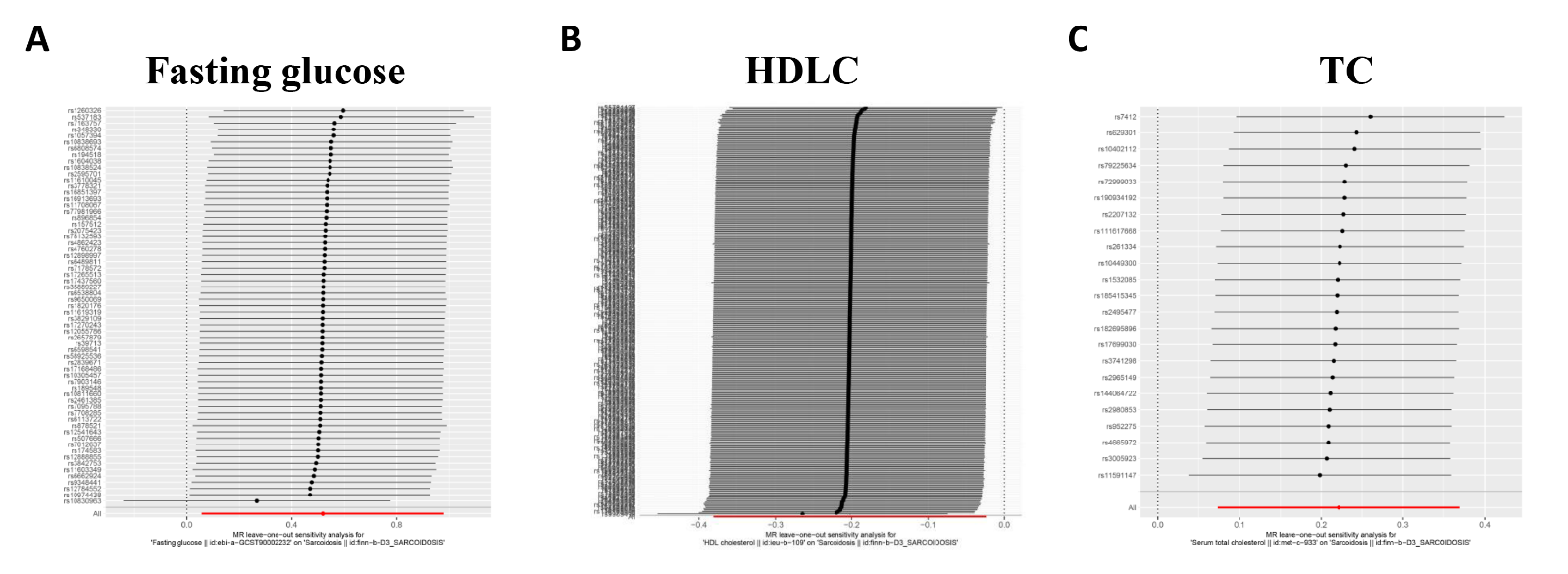


**Fig. S4.** Leave-one-out sensitivity analysis of the MR results.

Shown are the leave-on-out analysis for fasting glucose (A), HDLC (B), and TC (C). MR, mendelian randomization. HDLC, high density lipoprotein cholesterol. TC, total cholesterol.

**Table S1.** Detailed information of studies included in mendelian randomization study

| **Phenotype** | **Abbreviation** | **Consortium/First author** | **Sample size** | **Ethnicity** | **Year** | **GWAS_ID** |
| --- | --- | --- | --- | --- | --- | --- |
| Sarcoidosis | - | FinnGen | 217,758 | European | 2021 | [finn-b-D3_SARCOIDOSIS](https://gwas.mrcieu.ac.uk/datasets/finn-b-D3_SARCOIDOSIS/) |
| Fasting glucose | FG | Chen | 200,622 | European | 2021 | ebi-a-GCST90002232 |
| High density lipoprotein cholesterol | HDLC | UK Biobank/Richardson | 403,943 | European | 2020 | ieu-b-109 |
| Low density lipoprotein cholesterol | LDLC | UK Biobank/Richardson | 440,546 | European | 2020 | ieu-b-110 |
| Total cholesterol | TC | Kettunen | 21,491 | European | 2016 | met-c-933 |
| Total triglycerides | TG | Kettunen | 21,545 | European | 2016 | met-c-934 |

**Table S2.** Distribution of lesions confirmed by pathological biopsy in sarcoidosis

| **Subtype** | **N (%)** |
| --- | --- |
| **Total** | 162 |
| **Single-site lesions** | 129 (79.6%) |
| Bronchus and lungs | 38 (23.5%) |
| Lymph nodes | 81 (50.0%) |
| Mediastinal | 54 (33.3%) |
| Axillary | 2 (1.2%) |
| Cervical | 22 (13.6%) |
| Elbow | 1 (0.6%) |
| Inguinal | 2 (1.2%) |
| Skin | 10 (6.2%) |
| **Multi-site lesions** | 33 (20.4%) |
| Bronchus and lungs + Mediastinal lymph nodes | 13 (8.0%) |
| Bronchus and lungs + Cervical lymph nodes | 6 (3.7%) |
| Bronchus and lungs + Skin | 1 (0.6%) |
| Bronchus and lungs + Mediastinal lymph nodes + Cervical lymph nodes | 1 (0.6%) |
| Bronchus and lungs + Mediastinal lymph nodes + Axillary lymph nodes | 1 (0.6%) |
| Mediastinal lymph nodes + Cervical lymph nodes | 7 (4.3%) |
| Mediastinal lymph nodes + Pleura | 1 (0.6%) |
| Skin + Mediastinal lymph nodes | 1 (0.6%) |
| Skin + Cervical lymph nodes | 1 (0.6%) |
| Cervical lymph nodes + Inguinal lymph nodes | 1 (0.6%) |

**Table S3.** Collinearity diagnostics for multivariable logistic regression analysis before propensity score matching

|  | **t** | **Sig.** | **Tolerance** | **VIF** |
| --- | --- | --- | --- | --- |
| **Sex** | -0.427 | 0.670 | 0.469 | 2.130 |
| **Age** | -0.011 | 0.991 | 0.702 | 1.424 |
| **Weight** | -2.709 | 0.007 | 0.596 | 1.677 |
| **Smoking history** | 0.245 | 0.807 | 0.734 | 1.362 |
| **Hypertension** | -1.163 | 0.245 | 0.828 | 1.207 |
| **Uveitis** | 2.13 | 0.033 | 0.952 | 1.051 |
| **Fasting glucose** | 4.002 | 0 | 0.817 | 1.224 |
| **TC** | 8.608 | 0 | 0.275 | 3.633 |
| **TG** | -0.722 | 0.470 | 0.599 | 1.67 |
| **LDLC** | -2.649 | 0.008 | 0.352 | 2.843 |
| **HDLC** | -6.164 | 0 | 0.639 | 1.564 |
| **Blood potassium** | -3.121 | 0.002 | 0.879 | 1.138 |
| **Blood calcium** | 3.059 | 0.002 | 0.650 | 1.539 |
| **AST** | -1.188 | 0.235 | 0.777 | 1.286 |
| **GGT** | 1.845 | 0.065 | 0.501 | 1.994 |
| **ALP** | -1.154 | 0.249 | 0.536 | 1.865 |
| **ALB** | -4.173 | 0 | 0.617 | 1.622 |
| **Globulin** | 4.634 | 0 | 0.757 | 1.321 |
| **TBil** | 2.365 | 0.018 | 0.805 | 1.242 |
| **PLT** | 2.228 | 0.026 | 0.726 | 1.377 |
| **WBC** | -5.314 | 0 | 0.761 | 1.314 |

VIF, variance inflation factor. TC, total cholesterol. TG, total triglyceride. LDLC, low density lipoprotein cholesterol. HDLC, high density lipoprotein cholesterol. AST, aspartate amino transferase. GGT, gamma glutamyl transferase. ALP, alkaline phosphatase. ALB, albumin. TBil, total bilirubin. PLT, platelet. WBC, white blood cell.

**Table S4.** Collinearity diagnostics for multivariable logistic regression analysis after propensity score matching

|  | **t** | **Sig.** | **Tolerance** | **VIF** |
| --- | --- | --- | --- | --- |
| **Fasting glucose** | 1.971 | 0.050 | 0.856 | 1.168 |
| **TC** | 5.53 | 0 | 0.333 | 3.005 |
| **TG** | -1.124 | 0.262 | 0.628 | 1.594 |
| **LDLC** | -0.903 | 0.367 | 0.412 | 2.426 |
| **HDLC** | -3.92 | 0 | 0.648 | 1.543 |
| **Blood potassium** | -3.767 | 0 | 0.902 | 1.108 |
| **AST** | -2.078 | 0.039 | 0.802 | 1.247 |
| **GGT** | 1.303 | 0.194 | 0.689 | 1.451 |
| **ALB** | -2.676 | 0.008 | 0.908 | 1.101 |
| **Globulin** | 4.114 | 0 | 0.896 | 1.116 |
| **TBil** | 1.888 | 0.060 | 0.880 | 1.137 |
| **UA** | 2.47 | 0.014 | 0.798 | 1.253 |

VIF, variance inflation factor. TC, total cholesterol. TG, total triglyceride. LDLC, low density lipoprotein cholesterol. HDLC, high density lipoprotein cholesterol. AST, aspartate amino transferase. GGT, gamma glutamyl transferase. ALB, albumin. TBil, total bilirubin. UA, uric acid.

**Table S5.** Heterogeneity and pleiotropy tests of the reverse mendelian randomization analysis

| **Test** | **Outcome** | **Method** | **Effect size** | ***P* value** |
| --- | --- | --- | --- | --- |
| Heterogeneity | Fasting glucose | Cochran’s Q test | 5.2 (Q_MR Egger_) | 0.160 |
|  |  |  | 5.3 (Q_IVW_) | 0.257 |
|  | HDLC | Cochran’s Q test | 111.5 (Q_MR Egger_) | 0.000 |
|  |  |  | 120.8 (Q_IVW_) | 0.000 |
|  | LDLC | Cochran’s Q test | 69.7 (Q_MR Egger_) | 0.000 |
|  |  |  | 74.7 (Q_IVW_) | 0.000 |
|  | TC | Cochran’s Q test | 3.6 (Q_MR Egger_) | 0.312 |
|  |  |  | 3.6 (Q_IVW_) | 0.467 |
|  | TG | Cochran’s Q test | 5.2 (Q_MR Egger_) | 0.160 |
|  |  |  | 5.3 (Q_IVW_) | 0.257 |
| Pleiotropy | Fasting glucose | MR-Egger regression | 0.001 (Egger regression intercept) | 0.931 |
|  | HDLC | MR-Egger regression | 0.015 (Egger regression intercept) | 0.593 |
|  | LDLC | MR-Egger regression | 0.012 (Egger regression intercept) | 0.621 |
|  | TC | MR-Egger regression | 0.003(Egger regression intercept) | 0.954 |
|  | TG | MR-Egger regression | -0.014 (Egger regression intercept) | 0.792 |

MR, Mendelian randomization. IVW, inverse variance weighted. HDLC, high density lipoprotein cholesterol. LDLC, low density lipoprotein cholesterol. TC, total cholesterol. TG, total triglyceride.
